# Application of metagenomic next-generation sequencing technique for diagnosing a specific case of necrotizing meningoencephalitis caused by human herpesvirus 2

**DOI:** 10.1515/biol-2022-0464

**Published:** 2022-09-14

**Authors:** Xin Li, Jing Li, Yawei Shi, Guode Wu, Manxia Wang, Ye Zhang, Han Xia

**Affiliations:** Department of Neurology, Second Hospital of Lanzhou University, Lanzhou, China; Department of Scientific Affairs, Hugobiotech Co., Ltd., Beijing, China

**Keywords:** human herpesvirus 2, necrotizing meningoencephalitis, metagenomic next-generation sequencing, cerebrospinal fluid, case report

## Abstract

Reactivation of latent human herpesvirus 2 (HHV-2) can cause spontaneous recovering aseptic meningitis and recurrent meningitis in adults, but it rarely affects the brain parenchyma to cause encephalitis. Here, we report the case of a 37-year-old male patient admitted to our hospital due to fever with a progressive headache for 3 days and paroxysmal episodes of unconsciousness for 1 day. Brain magnetic resonance imaging (MRI) revealed viral meningoencephalitis. Then, metagenomics next-generation sequencing (mNGS) was applied, which detected 12,024 unique sequences of HHV-2 in cerebrospinal fluid (2022), indicating HHV-2 encephalitis. After antiviral treatment, the patient’s symptoms improved, and he was discharged. During the 1-month follow-up, the patient recovered without any new symptoms, but a brain MRI revealed significant atrophy of the original foci. The patient was finally diagnosed with HHV-2 necrotizing meningoencephalitis, which is extremely rare. mNGS helped with the clinical diagnosis and strengthened our understanding of HHV-2 infections in the central nervous system.

## 1 Introduction

Human herpesvirus 2 (also called HHV-2 or HSV-2) infection of the central nervous system can be primary or due to reactivation of latent virus [1,2]. Primary infection involving the central nervous system is more common in newborns and often causes multifocal or focal lesions of the temporal lobe, brainstem, or cerebellum [3,4]. Reactivation of the latent virus can lead to spontaneous recovery of aseptic meningitis and recurrent meningitis in adults, accounting for 1.6–6.5% of adult herpes simplex meningitis, but it rarely affects brain parenchyma to cause encephalitis [5].

Metagenomics next-generation sequencing (mNGS) is currently applied in many clinical practices and has a high sensitivity and specificity in the diagnosis of various pathogens, especially viruses [6,7]. In this study, mNGS was used to detect the pathogen. In this case, HHV-2 meningoencephalitis complicated by rapid atrophy of the temporal lobe was finally confirmed, which is an extremely rare complication of HHV-2 infection.

### 1.1 Case presentation

A 37-year-old male patient was admitted to the Second Hospital of Lanzhou University on 23rd July 2019 due to “fever accompanied by progressively deteriorating headache for 3 days and paroxysmal unconsciousness for 1 day.” The patient had a history of “herpes zoster” 6 months ago. He developed fever without obvious predisposing factors 3 days before admission. His body temperature was 38.4–39.4°C, accompanied by a headache, mainly a mild intermittent throbbing pain in the temporal region. The symptoms aggravated the next day. The headache spread to the occipital region and the back of the neck and lasted longer. Meanwhile, the patient experienced mild fatigue and sore muscles that were unresponsive to ibuprofen sustained-release capsules. On the morning of 22nd July, the headache became significantly aggravated and more persistent, accompanied by nausea and vomiting of stomach contents two times. The patient suddenly lost consciousness that night, accompanied by involuntary movement of the left limbs, and regained consciousness 0.5 min later. When visiting our hospital on 23rd July, the patient lost consciousness again, accompanied by involuntary movement of the left limbs, and regained consciousness 1 min later.

**Figure 1:**
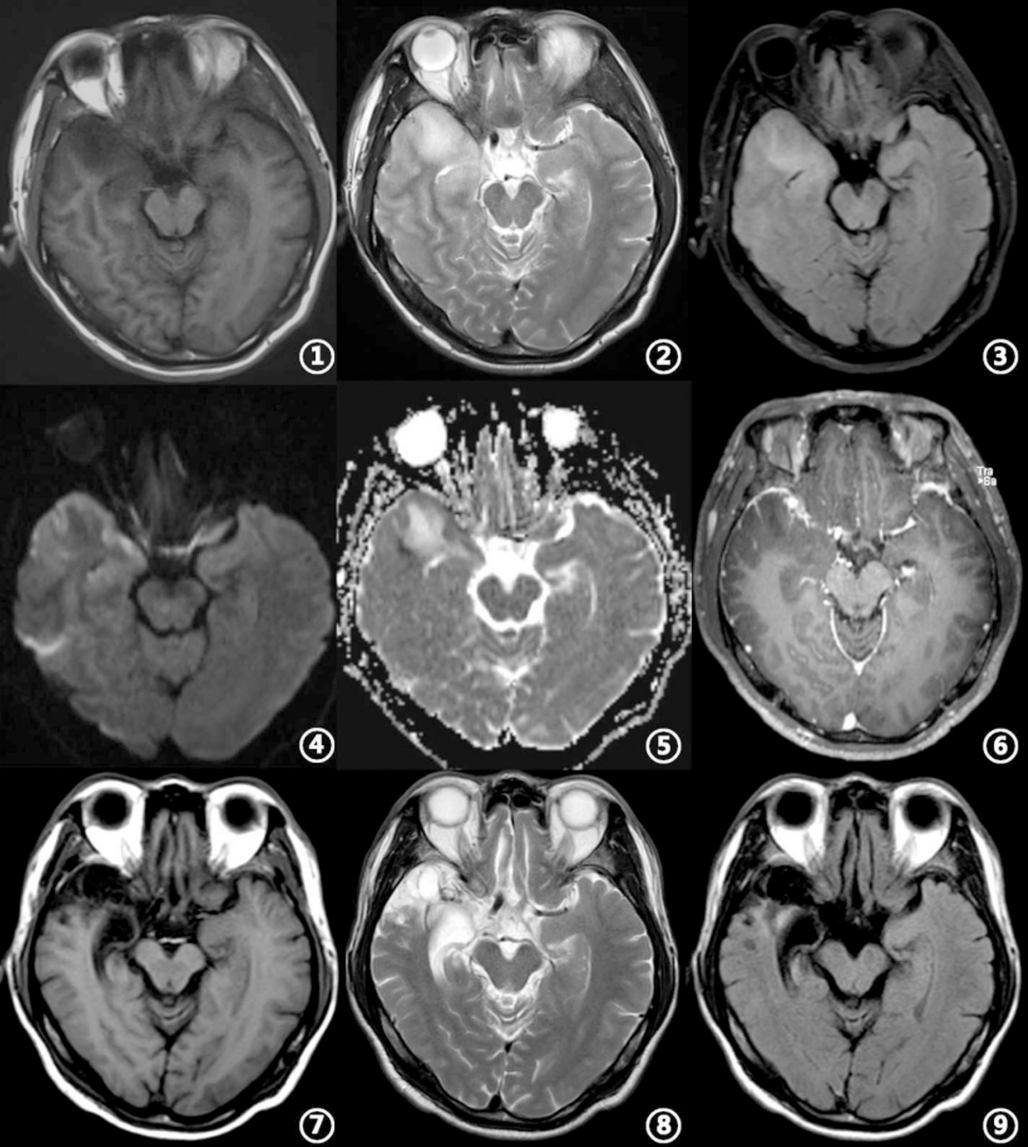
Head MRI results of the patient. ①–⑥ was on admission, and ⑦–⑨ was 1 month later. ① and ② Patchy long T1 signal and slightly longer T2 signal in the right frontal, temporal, and insular lobes; ③–⑤ High signals in Flair and DWI lesion areas, and slightly higher signal in ADC area; ⑥ Patchy enhanced shadows in some lesion areas, and the dura mater adjacent to these lesion areas was enhanced and thickened; ⑦–⑨ Patchy long T1 and T2 signals in the right frontal, temporal pole, and insular lobes, local atrophic, necrotic, and softening foci, and widening of the right temporal horn.

**Figure 2:**
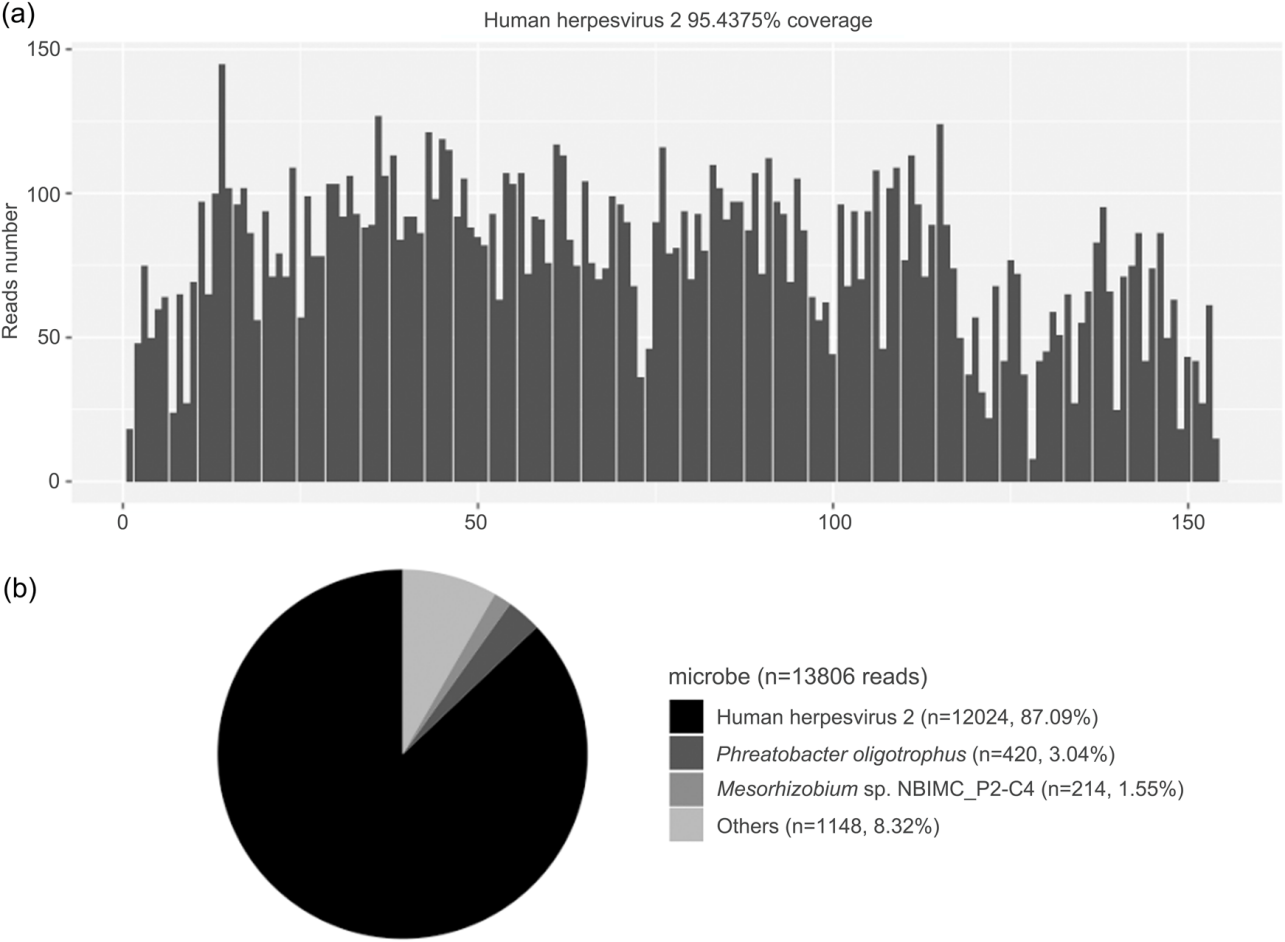
mNGS results of the patient. (a) The DNA reads positions and coverage of HHV-2 detected by mNGS. The coverage of HHV-2 detected by mNGS was 95.44%. (b) The content of detected microbes by mNGS. A total of 12,024 unique reads (87.09%) of HHV-2 were sequenced.


Physical examination on admission showed a body temperature of 38.2°C, a pulse-93 beats/min, a respiratory rate of 23 times/min, and a blood pressure of 124/82 mmHg. Scattered rice-like scabs caused by previous herpes zoster were observed in the sixth left intercostal space, with old skin lesions and fusion, but without redness or ulceration. The nervous system examination showed consciousness and a poor mental state without obvious abnormalities involving high-level cortical functions or cranial nerves. The muscle strength of the four limbs was graded as 4+. The muscle tension was normal, and tendon reflexes were present without any pathological signs. The neck was rigid, and the mentosternal distance was four fingers. Kernig sign+ and Brudzirnki sign+ were observed. Blood routine on admission plus C-reactive protein (CRP) showed white blood cell (WBC) counts at 9.5 × 10^9^/L, neutrophil ratio (NE%) of 0.51, lymphocyte ratio (LY%) of 0.49, and CRP of 21 mg/L. The rest of the laboratory tests showed no abnormalities (Table 1). Electroencephalography (EEG) showed rhythmic emission of focal slow waves in the right frontal and temporal regions at 2–6 cycles per second, spreading to all leads. Preliminary diagnosis on admission indicated central nervous system infection of suspected viral meningoencephalitis and secondary epilepsy. Acyclovir 0.5 g/8 h, intravenously guttae (ivgtt), dexamethasone 10 mg, ivgtt, and carbamazepine 200 mg orally twice daily. were administered. Lumbar puncture was subsequently performed with the cerebrospinal fluid (CSF) intracranial pressure of 210 mmH_2_O, WBC counts of 145 × 10^6^/L, mononuclear cell ratio (MN%) of 97.7, and proteins of 0.84 g/L. The rest examinations showed no abnormalities. In addition, 4 mL of CSF was collected for Pathogen Capture Engine Sequence (PACEseq) mNGS (Hugobiotech, Beijing, China) to determine the pathogen. In the meantime, autoimmune encephalitis antibody, infectious disease (including *Toxoplasma*, rubella virus, cytomegalovirus, herpes simplex virus, and other pathogenic microorganisms), rheumatism, respiratory tract 9-items, and tuberculosis antibody were all tested but showed negative. Brain magnetic resonance imaging (MRI) on July 25th showed patchy long T1 signal and slightly longer T2 signal in the right frontal, temporal, and insular lobes. While on diffusion-weighted imaging (DWI) and fluid-attenuated inversion recovery (FLAIR) sequences, high signals and patchy enhanced shadows in the lesion areas were also observed, and the dura mater adjacent to the lesion areas was enhanced and thickened (Figure 1). On July 26th, the mNGS result came out, and a total of 12,024 unique sequences of HHV-2, with a coverage of 95.44% were detected (Table 1, Figure 2), indicating HHV-2 meningoencephalitis. On 28th July, the patient’s autoimmune encephalitis-related antibody was reported negative. After 21 days of antiviral treatment, the re-examination of the CSF showed intracranial pressure of 120 mmH_2_O, WBC counts of 4 × 10^6^/L, and MN% of 100. No abnormalities were found for the rest items. The patient was discharged and given acyclovir and carbamazepine. One month later, an EEG re-examination on 15th September showed normal results. Head MRI showed irregular long T1 and T2 signal shadows in the right temporal pole. Low signals in FLAIR lesions and patchy high signals in the periphery were also detected by DWI. When compared with previous findings, temporal lobe and hippocampus atrophy adjacent to the lesions were observed. Local necrosis and softening foci were formed, and the right temporal horn was widened (Figure 1). After discharge from the hospital, the patient felt significant memory loss, without other obvious discomforts.

**Table 1:** The clinical tests, laboratory tests, and mNGS results of the patient

Time of sample collection	Time of test report	Items	Result
23rd July	23rd July	Blood routine plus CRP	WBC 9.5 × 10^9^/L (NE% 0.51, LY% 0.49), CRP 21 mg/L
23rd July	23rd July	CSF test	WBC 145 × 106/L (MN%, 97.7), Proteins 0.84 g/L
23rd July	24th July	Infectious disease, rheumatism, respiratory tract 9-items, and tuberculosis antibody	Negative
23rd July	26th July	CSF mNGS	HHV-2 (12,024 unique reads, coverage of 95.44%)
23rd July	28th July	AE antibody	Negative
13th August	13th August	CSF test	Negative

**Informed consent:** Informed consent has been obtained from all individuals included in this study.
**Ethical approval:** The research related to human use has complied with all the relevant national regulations, institutional policies and in accordance with the tenets of the Helsinki Declaration, and has been approved by the ethical review committee of Guangdong Provincial Hospital of Integrated Traditional Chinese and Western Medicine.

### 1.2 mNGS detection

QIAamp DNA Micro Kit (QIAGEN, Hiden, Germany) was used to extract the DNA of the CSF sample from the patient. The library was constructed using QIAseq™ Ultralow Input Library Kit for Illumina (QIAGEN, Hiden, Germany) and quality assessed using Qubit (Thermo Fisher, Waltham, USA) and Agilent 2100 Bioanalyzer (Agilent Technologies, Palo Alto, USA). NextSeq 550 platform (Illumina, San Diego, USA) was applied to sequence the DNA of the qualified library. Adapter, short, low-quality, and low-complexity read as well as human DNA read mapping to the human reference database (hg38) were removed from the raw data of each library. The remaining reads were aligned to the Microbial Genome Databases (ftp://ftp.ncbi.nlm.nih.gov/genomes/) using Burrows–Wheeler Aligner software. The reads number and coverage of each detected microbe were reported.

## 2 Discussion

HHV-2 belongs to α-herpesvirinae, and the human is its only host. HHV-2 is often associated with external genital infections. This sexually transmitted disease is second only to HIV infection, and oral lesions may occur occasionally [1,2]. Central nervous system infections caused by HHV-2 can be primary or due to the reactivation of the latent virus. Primary infection of HHV-2 involving the central nervous system is more common in newborns [3,5]. It often causes multifocal lesions or confined lesions in the temporal lobe, brain stem, or cerebellum, and the involvement of deep gray matter structures, accompanied by hemorrhage, is observed in 57% of the cases [3,5]. Previous studies showed that HHV-2 was a common cause of spontaneously healed aseptic meningitis and recurrent meningitis in adults [4,8]. This is mainly caused by reactivation of the latent virus and is more commonly seen in the elderly and/or immunosuppressed individuals, accounting for 1.6–6.5% of adult herpes simplex meningitis [9]. However, it rarely affects brain parenchyma and causes encephalitis. In 1997, Dennett et al. [10] retrospectively analyzed 64 cases of herpes simplex virus encephalitis and found that only one case was caused by HHV-2. In 2008, Danish scholar Omland et al. [11] analyzed 49 patients with HHV-2 infection involving the central nervous system and found only six cases with encephalitis manifestations. Unlike the reported cerebellar brainstem inflammation and myelitis caused by HHV-2 [12], HHV-2 herpes encephalitis resulting in brain atrophy within a short period of time has never been reported. In this case report, the patient manifested HHV-2 infection of the right temporal and insular lobes, accompanied by enhancement adjacent to the meninges, which was consistent with the imaging characteristics of meningoencephalitis. One month later, the re-examination imaging showed significant atrophy and necrosis of the original right temporal and insular lobe lesions. This imaging finding was a typical manifestation of herpes simplex virus type 1 (also called HHV-1 or HSV-1) meningoencephalitis and has never been reported in HHV-2 meningoencephalitis cases in the past.

Since the application of mNGS in the diagnosis of central nervous system infections in 2014 [13], it has become a hotspot in the field of neuroinfection. mNGS can quickly and accurately detect almost all pathogens, especially for cases that are hard to be confirmed by routine examinations. It plays an important role in clinical diagnosis [14]. In this study, the patient had a history of herpes zoster prior to developing encephalitis, zoster encephalitis could not be ruled out. mNGS can differentiate varicella-zoster virus (HHV-3) from other herpes viruses [14]. A large number of HHV-2 unique sequences (12,024 reads) were detected by mNGS in this case, indicating HHV-2 necrotizing meningoencephalitis rather than zoster encephalitis, which had never been found before. mNGS helped the clinical diagnosis and strengthen our understanding of HHV-2 infections in the central nervous system.
